# Supplementary material for: Coherent long-distance displacement of individual electron spins
Source: Nat Commun. 2017 Sep 11;8:501. doi: 10.1038/s41467-017-00534-3 (PMC5593884; doi:10.1038/s41467-017-00534-3)
Supplement: Supplementary file 1 — Supplementary Information [file 41467_2017_534_MOESM1_ESM.pdf]

Description of Supplementary Files

File Name: Supplementary Information
Description: Supplementary Figures, Supplementary Notes and Supplementary References

File Name: Peer Review File

Supplementary Note 1. Circular triple-dot in the isolated configuration

In the main text, the manipulation and the control of two electrons in the isolated configuration of a circular triple-dot system is described. In this section, we provide an electrostatic simulation of the potential experienced by a single electron (see Supplementary Fig. 1a), and we describe in details how to reach the isolated configuration and the stability diagram corresponding to different numbers of electrons.

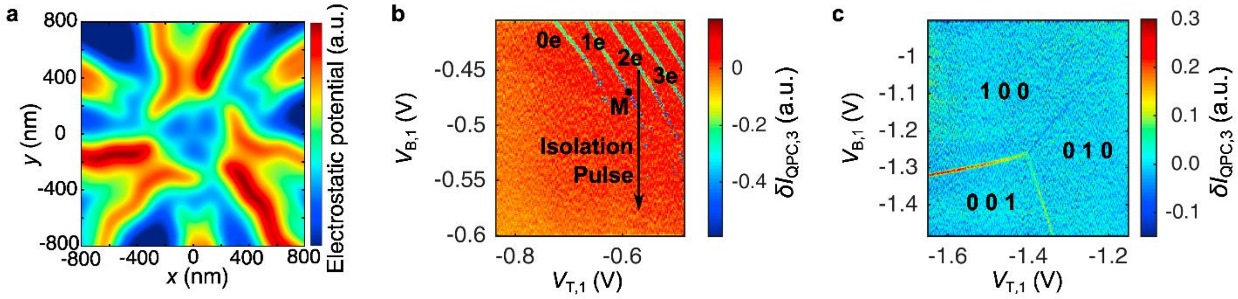

Supplementary Figure 1. Circular triple quantum dot in the isolated regime. a, Electrostatic potential experienced by the electron in a working configuration showing the three quantum dots in a circular configuration. The calculation follows the procedure presented in ref 31. The distance between two dots is estimated to be 110 nm. **b**, Stability diagram of the bottom quantum dot close to the isolated regime. Derivative $\delta I_{QPC,3}$ of $I_{QPC,3}$ when the system is scanned with the gates $V_{B,1}$ and $V_{T,1}$. The charge degeneracy lines are vanishing because the tunnelling time to the reservoir is becoming progressively longer as $V_{B,1}$ becomes more negative. The loading positions for one, two, three electrons and the spin measurement position are indicated with 1e, 2e, 3e and M, respectively. **c**, Isolated stability diagram for the case of one electron initialized in the system. Derivative $\delta I_{QPC,3}$ of $I_{QPC,3}$ when the system is scanned in the one-electron isolated configuration with the gates $V_{B,1}$ and $V_{B,2}$. The dot occupation numbers are given in the graph following the notation of Fig. 1b.

In the isolated configuration, we are able to characterize the triple-dot system with a fixed number of electrons over a wide range of gate voltages. Following the procedure presented in ref 1 and 2, it is indeed possible to load first the bottom dot with the desired

number of electrons and then rapidly promote them into the isolated position with a microsecond pulse mainly applied on the blue gates of Fig. 1a (see Supplementary Fig. 1b). Finally, the system is scanned from that position to reconstruct a stability diagram of the isolated triple dot. Supplementary Figure 1c (Figure 1b) shows the observed stability diagram with the overall electron number fixed to one (two). They are characterized by three (six) distinct charge configurations separated by inter-dot degeneracy lines. These different charge configurations are the only possible charge states with a fixed number of electrons loaded in the isolated configuration[1, 2].

Supplementary Note 2. Tunnel-rate spin read-out in the isolated configuration and its fidelity

To perform spin read-out of the two-electron spin states in the isolated configuration, the two electrons are brought back into the bottom dot in a configuration where the electrons can be exchanged with the reservoir[1]. In this section, a detailed description of the spin read-out procedure and an evaluation of the spin read-out fidelity is given.

To probe the spin dynamics of the electrons in the triple-dot system, a spin readout protocol compatible with the electron displacement procedure has been implemented. The principle to detect electron spin states in a single quantum dot coupled to a lead is well established[1, 3, 4]. It relies on the engineering of a spin-dependent tunnel process from the dot to the reservoir to convert spin into charge information. To perform the single shot readout of a two-electron spin state, we take advantage of the difference in tunnel-rates for singlet (Γ_S) and

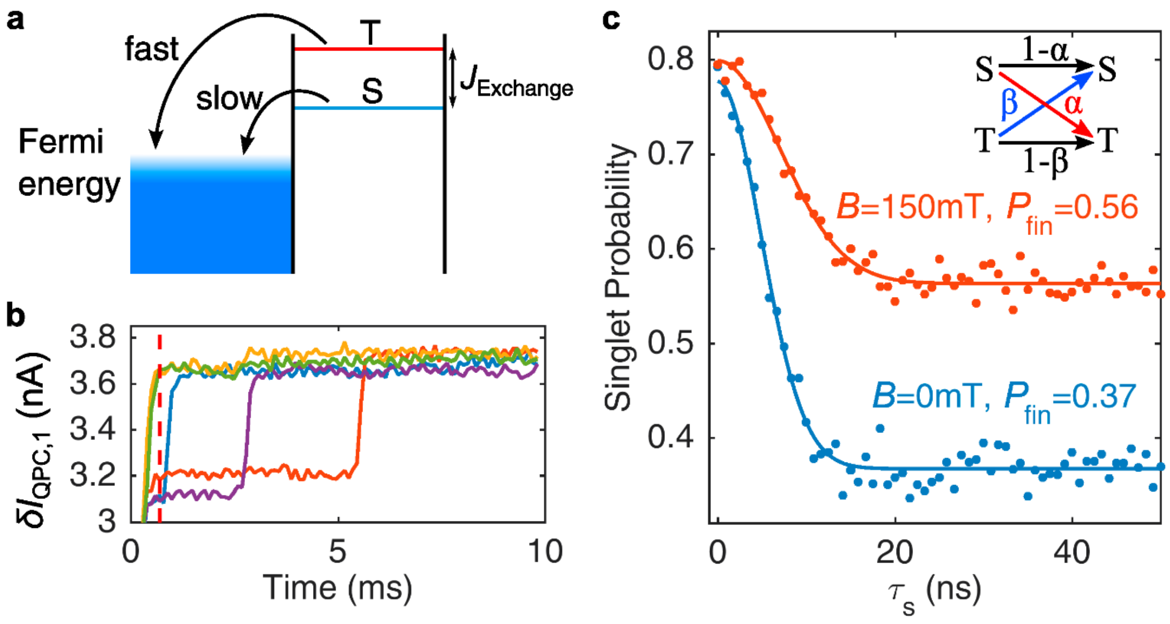

Supplementary Figure 2 Tunnel-rate dependent spin readout. a, Principle of the spin read-out based on the difference of the tunnel-rate to the reservoir between the singlet S and the triplet T states. **b,** Typical single-shot current time-traces used to distinguish S and T states. An electron tunnelling out of the dot results in a current jump from the two-to-one electron charge configuration. The red dashed line indicates the time-threshold condition for triplet identification. **c,** Singlet probability as a function of τ_s measured in the isolated position where hyperfine interaction is dominant at zero (blue) and 150 mT (red) external magnetic field. A Gaussian fit (solid lines) reveals a mixing time equal to 6 ns and 10 ns respectively. The final spin mixing values P_{fin} allow us to estimate the errors α and β in the detection of the singlet and the triplet states according to the error scheme shown in the inset.

triplet states (Γ_T) at the measurement position M in Supplementary Fig. 1b where one electron is allowed to tunnel out of the dot (see Supplementary Fig. 2a and b). We estimate the ratio Γ_T/Γ_S to be 10 in our experiment fixed by the shape of the dot. However, the scheme used to displace the electrons requires working with the triple-dot system in an isolated configuration, where no exchange of electrons is possible between the dot and the reservoir[1]. For this reason, we have to bring back the dot system to the measurement position at a µs-timescale (much faster than the spin relaxation time) to infer the electron spin state in the isolated configuration.

To quantify the fidelity of the complete spin read-out procedure, we have analysed the evolution of the singlet probability as a function of the time τs where the electrons are separated at different magnetic fields (see Supplementary Fig. 2c). At zero magnetic field, all the three triplet states should mix with the singlet state whereas only T_0 mixes with the singlet state at 100 mT. The data are fitted with a Gaussian decay. The observed loss of coherence is explained by the fluctuating effective magnetic field difference between the dots of 4 mT due to the hyperfine interaction. The initial and steady-state probability values of the decay curves allow extracting the measurement errors α and β for singlet and triplet respectively. The electron should start in singlet states for both magnetic field conditions whereas the proportion of singlet at the end of the mixing should be 0.5 at 0.15 T and 0.3 at 0 T[5]. Such an analysis gives us an average measurement fidelity of 1 - $(\alpha+\beta)/2$ = 80%.

Supplementary Note 3. Model of the triple dot system with two electrons

A spin map procedure has been implemented to identify where spin-mixing between the singlet and the triplet states occurs in the gate voltage space. Such a mixing is the result of a competition between the hyperfine and the exchange interactions. In a double dot[3], the level repulsion induced by tunnelling only affects the singlet states and results in an energy splitting $J_{exchange}$, the exchange energy (see Supplementary Fig. 2a), between singlet and triplet states. In this section, the three quantum dot system, circularly tunnel-coupled, with two electrons is modelled in order to simulate the result of the spin mixing map procedure.

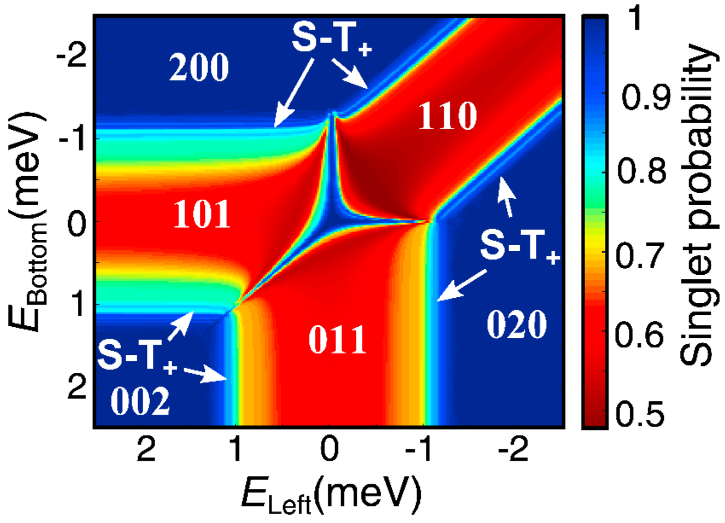

Supplementary Figure 3 Simulated spin mixing map for the triple dot with two electrons. Singlet probability after 50 ns of free evolution as a function of the energy of the left (E_{Left}) and bottom quantum dot (E_{Bottom}). The energy of the right quantum dot was chosen to be $E_{\mathrm{Right}} = 0$. The charge configuration is first inferred from the lowest energy eigenstates of the Hamiltonian and the appropriate singlet state is selected. Then a time evolution for the respective energy configuration is performed and the singlet probability is averaged over Gaussian distributed nuclear field values in each quantum dot. In this diagram, the tunnel-coupling energy is $t = 12$ µeV and the Zeeman energy splitting from the magnetic field is $E_Z = 4$ µeV.

In this simulation, the hyperfine interaction is modelled as an effective magnetic field B_n in each dot that is fluctuating between two realizations of the experiment with a standard deviation ΔB_n= 2.8 mT. The relevant parameters of the Hamiltonian are then the effective magnetic field in each dot, the tunnel-couplings between the dots assumed to be equal, and the chemical potential and the charging energy of each dot. In each gate voltage configuration, we plot the result of the 50-ns spin evolution starting from the singlet state with an averaging on the fluctuating effective magnetic field conditions. The results of the simulation is presented in Supplementary Fig 3. It reproduces qualitatively the main features of the measured spin map. In particular, an increase of the singlet population in the region where the two electrons are separated is observed when the system is close to the degeneracy between two dots, for

example the (1,1,0)-(1,0,1) crossing region. In a circularly coupled triple-dot, up to three tunnel-couplings are indeed contributing to the repulsion of the singlet states and an increase of the exchange interaction close to the separated degeneracy line is expected.

Supplementary Note 4. Comparison between Gaussian and exponential fit of the transfer data

In this section, we present a comparison of the Gaussian and exponential fitting procedures data presented in Fig. 3c (see Supplementary Fig. 4). It shows that the best agreement is obtained with an exponential law for the observed mixing between singlet and triplet as a function of the separation time in the case where the static phase is minimized.

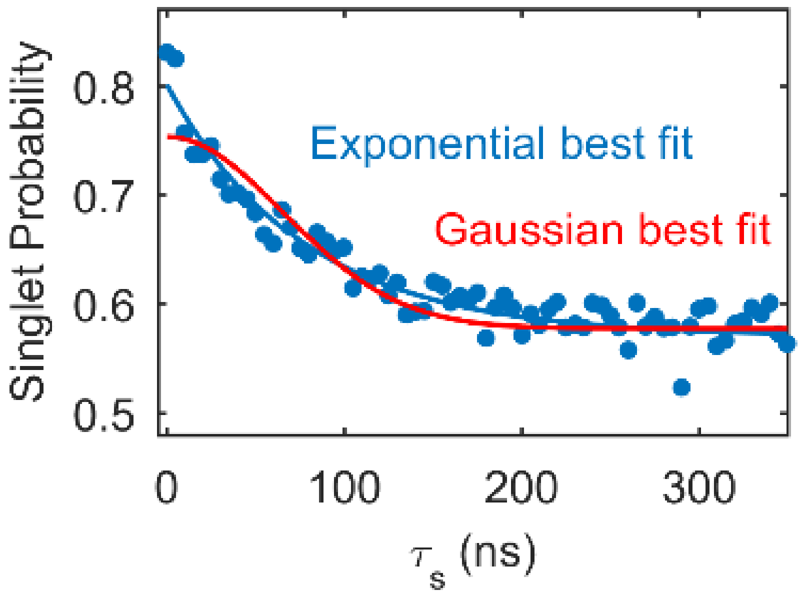

Supplementary Figure 4 Comparison of the Gaussian (red) and exponential (blue) fitting procedures data presented in Fig. 3c (plain blue circles).

Supplementary Note 5. Increase of the spin coherence time while the two electrons are moving at zero magnetic field

The increase in spin coherence time at a magnetic field of 200 mT is precisely analysed in the main text and a strong dependence of the spin coherence with magnetic field is shown.

In this section, we present the investigation of the impact of displacement on the spin mixing at zero magnetic field. We have implemented the same procedure as discussed in Fig. 2b and 3c at zero magnetic field. The resulting singlet probabilities as a function of the time τ_S where the electrons are separated are presented in Supplementary Fig. 5. Without displacing the electrons, the singlet probability is characterized by a Gaussian decay with a typical timescale close to 6 ns. Such a reduction of the spin coherence time at zero magnetic field is expected since all the three triplet states can be mixed with the singlet state due to hyperfine interaction and the three components of the effective magnetic field have to be taken into account[24]. With the electron displacement, an exponential decay of the singlet probability is observed with an increase of the spin coherence time by a factor of two.

We conclude that the spin-flip process is more efficient at zero magnetic field. In this situation, the electrons do not need an energy exchange with a reservoir to flip their spin. In the manuscript, two mechanisms were identified as the main sources of decoherence during electron displacement: spin-orbit and transverse hyperfine interactions. At zero field, the spin-orbit interaction results in a coherent evolution of the electron spin during its displacement on a fixed path[6, 7]. The singlet state is then expected to be preserved along the displacement. To be in agreement with the data, changes of the electron path due to the large microwave excitation

could result in a fast mixing of the singlet state with the triplet states. It is worth noting that the transverse hyperfine interaction is in essence a fluctuating coupling between the spin and the motion and, on the contrary to the spin-orbit interaction, path fluctuations are therefore not required to explain the fast mixing of the singlet state with triplet states.

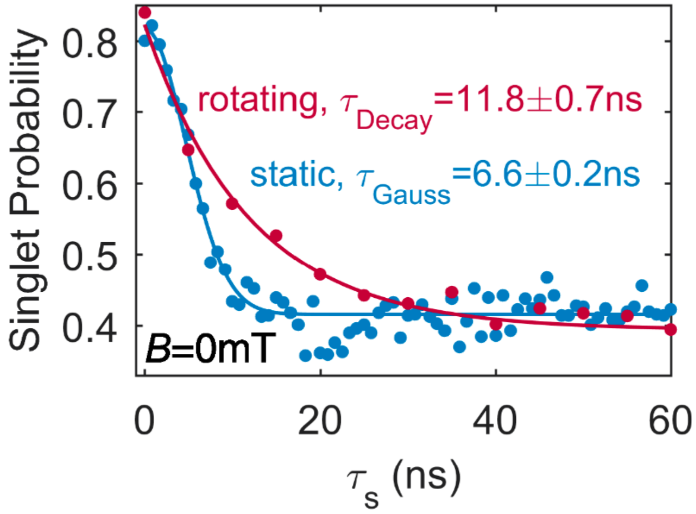

Supplementary Figure 5 Coherent spin displacement at zero magnetic field. Singlet probability as a function of the time τ_s for the case where the electrons are static in the (0,1,1) charge configuration (blue) and where they are rotating between separated charge configurations with τ_r = 1.7 ns (red). The data are fitted with a Gaussian decay with a characteristic time T_2^* for the static procedure. The case for rotational movement shows better agreement with an exponential decay with a characteristic time τ_{Decay} and shows a significantly longer decay time constant.

Supplementary Note 6. Influence of the tunnel-coupling on the displacement-induced spin coherence time

In the main text, the presented results have been mostly obtained for a specific tunnel-coupling between the dots. In this section, the spin coherence time after displacement for different tunnel-couplings between the dots is analysed. The strength of the tunnel-couplings can be changed by controlling the potential of the red gates in Fig. 1a. It is worth noting that

the tunnel coupling is not affected by the perpendicular magnetic field in the range (0-200mT) used in the experiment. The tunnel coupling can directly be witnessed on the spin mixing maps in Supplementary Fig. 6b-d: for decreasing tunnel-coupling, the separation between the three mixing regions is progressively vanishing. We observe that the exchange interaction at the single-electron crossing between two dots when the electrons are separated is reduced and becomes negligible in comparison with the hyperfine interaction. Moreover, the separation in gate voltage space between the S-T_0 mixing region and the S-T+ crossing lines is progressively reduced until it vanishes completely. Such observations are in agreement with a progressive reduction of the singlet level repulsion induced by the tunneling process and are consistent with a reduction of the tunnel-couplings between the dots. Moreover, the observed separations in gate voltage between the S-T_0 mixing regions are very similar (see Supplementary Fig. 6b) which is an indication that the tunnel-couplings between the dots are similar. Even though we observe clear differences in the spin mixing map as discussed in the previous paragraph, no significant change in the increase of the spin coherence time is observed when displacing the electrons (see Supplementary Fig. 6a). We attribute the slight difference in the measured spin coherence times to a change in the path of the electron during its displacement due to the altered gate voltage configuration. Such a dependence is expected to change the extracted constant N_{Decay} in Fig. 4a. It is worth noting that the displacement data presented in Fig. 4 (Fig. 2, 3 and 5) correspond to the gate configuration of Supplementary Fig. 6b (6c). We therefore conclude that the tunnelling strength is not strongly affecting the spin coherence as long as it is sufficient to allow transfer between the dots.

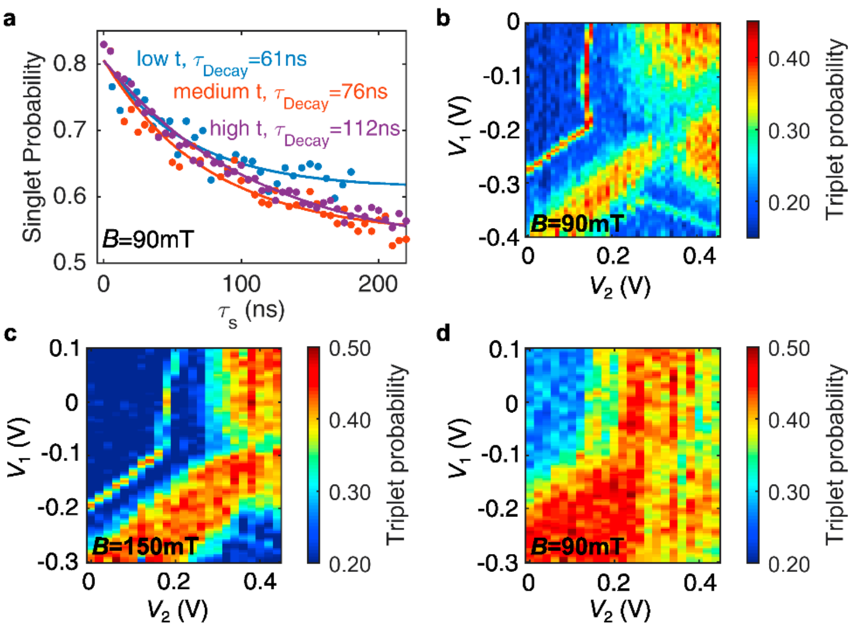

Supplementary Figure 6 Influence of the tunnel-coupling on the spin dynamics in the triple quantum dot. a, Singlet probability as a function of the time τ_S for the case where the electrons are rotating between separated charge configurations corresponding to the spin map presented in b (violet), c (orange) and d (blue). The data are fitted with an exponential decay with a characteristic time τ_{Decay}. **b, c and d,** Spin mixing maps for decreasing tunnel-coupling between dots. Singlet probability after a 50-ns pulse on V_1 and V_2.

Supplementary Note 7. Influence of the displacement geometry on the spin coherence time

In the manuscript, the increase of spin coherence is interpreted as a consequence of the electron displacement in a moving quantum dot before and after the tunnelling process. The demonstrated mechanism does therefore not require a particular geometry of the displacement to result in an increase of the spin coherence time. In this section, we provide additional measurements demonstrating that indeed a similar increase for a different transport geometry is observed.

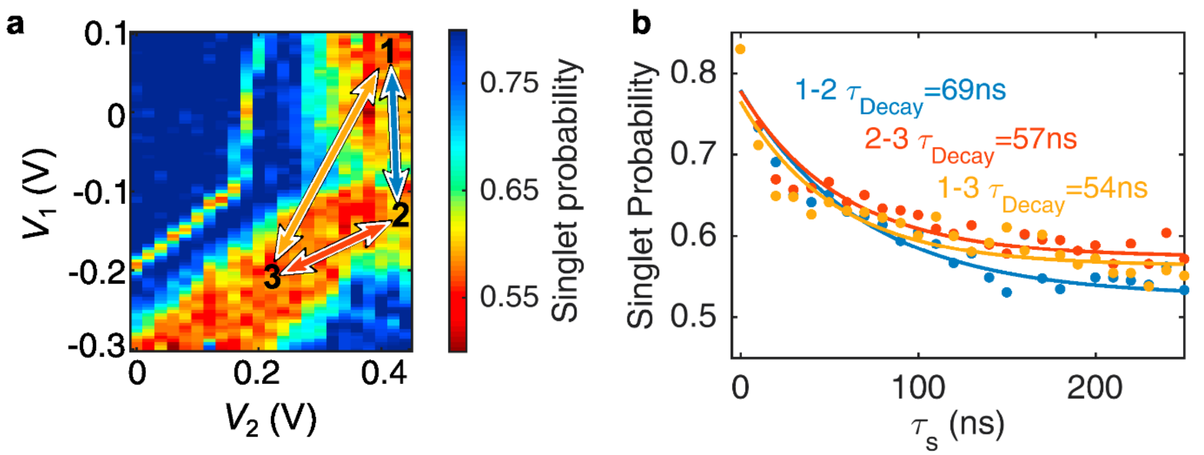

Supplementary Figure 7 Influence of the displacement geometry on the spin coherence time. **a,** Reproduction of the spin mixing map presented in Fig. S5c with colored arrows indicating the three possible single electron transfers. The system is repeatedly pulsed in between two of the three possible charge configurations to induce arbitrary long displacements of single electrons between two dots. **b,** Singlet probability as a function of the time τ_S for the case where one electron is displaced between two dots. The time τ_r spent in each charge configuration during the electron displacement is 2.5 ns, and a magnetic field of 150 mT is applied. A colour code correspondence between the arrows in a and the data in b is used. The data are fitted with an exponential decay with a characteristic time τ_{Decay}.

First, we have checked that the rotation direction of the displacement in the triple-dot system was not relevant. In the manuscript, the electrons were rotated anticlockwise along the circular path depicted in Fig. 3. No change of the spin coherence time was observed by rotating clockwise or by alternating clockwise and anticlockwise rotations (data not shown).

Second, we have analysed the situation where only one of the two electrons was displaced between two dots. In comparison with the procedure presented in Fig. 3, we only move one of the electrons back and forth between two static dot configurations after separating the two electrons. The resulting singlet probabilities as a function of the time τ_S where the electrons are separated are presented in Supplementary Fig. 7b. They show similar spin dynamics to the case where both electrons are displaced in a circular geometry. Such an observation rules out a scenario where the influence of the nuclei has been cancelled due to

electron displacement. Indeed, in a circular displacement, both electrons are experiencing exactly the same effective magnetic fields and as a consequence S and T_0 are not expected to mix anymore[8].

Supplementary References:

1. Bertrand, B. *et al*. Quantum manipulation of two-electron spin states in isolated double quantum dots. *Physical Review Letters* **115**, 096801 (2015).
2. Flentje, H. *et al*. A linear triple dot in the isolated configuration *Applied Physics Letters* **110**, 233101 (2017).
3. Hanson, R., Petta, J. R., Tarucha, S., Vandersypen, L. M. K. Spins in few-electron quantum dots. *Reviews of Modern Physics* **79**, 1217-1265 (2007).
4. Hanson, R. *et al.* Single-shot readout of electron spin states in a quantum dot using spin-dependent tunnel rates. *Physical Review Letters* **94**, 196802 (2005).
5. Merkulov, I. A., Efros, A. L., Rosen, M. Electron spin relaxation by nuclei in semiconductor quantum dots. *Physical Review B* **65**, 205309 (2002).
6. Huang, P., Hu, X. Spin qubit relaxation in a moving quantum dot. *Physical Review B* **88**, 075301 (2013).
7. C. Echeverría-Arrondo and E. Ya. Sherman, Relaxation of flying spin qubits in quantum wires by hyperfine interaction. *Phys. Rev. B* **87**, 081410(R) (2013).
8. Drummond, D., Pryadko, L. P., Shtengel, K. Suppression of hyperfine dephasing by spatial exchange of double quantum dots. *Physical Review B* **86**, 245307 (2012).
